# Human Dental Pulp-Derived Mesenchymal Stem Cell Potential to Differentiate into Smooth Muscle-Like Cells In Vitro

**DOI:** 10.1155/2021/8858412

**Published:** 2021-01-22

**Authors:** Jinhee Ha, Dinesh Bharti, Young-Hoon Kang, Sang-Yeob Lee, Seong-Ju Oh, Saet-Byul Kim, Chan-Hee Jo, Jang-Ho Son, Iel-Yong Sung, Yeong-Cheol Cho, Gyu-Jin Rho, Jeong-Kil Park

**Affiliations:** ^1^Department of Dentistry, Ulsan University Hospital, University of Ulsan College of Medicine, Ulsan, Republic of Korea; ^2^Department of Theriogenology and Biotechnology, College of Veterinary Medicine and Research Institute of Life Science, Gyeongsang National University, Jinju, Republic of Korea; ^3^Department of Oral and Maxillofacial Surgery, Changwon Gyeongsang National University Hospital, Gyeongsang National University School of Medicine, Jinju, Republic of Korea; ^4^Department of Conservative Dentistry, School of Dentistry, Pusan National University, Yangsan, Republic of Korea

## Abstract

Previous studies have shown that mesenchymal stem cells (MSCs) derived from various tissue sources can be differentiated into smooth muscle-like cells (SMLCs) in vitro. In this paper, dental pulp-derived mesenchymal stem cells (DPSCs) were evaluated for their differentiation ability towards smooth muscle-like cells (SMLCs) under the effect of widely used cytokines (TGF-*β*1 and PDGF-BB) with special focus on different culturing environments. For this purpose, both the commercially used culturing plates (Norm-c) and 0.1% gelatin-precoated (Gel-c) plates were used. Isolated cells displayed plastic adherence, pluripotency and cell surface marker profiling, and adipogenic and osteogenic differentiation potential with lineage specific marker expression. Differentiated cells induced under different culturing plates showed successful differentiation into SMLCs by positively expressing smooth muscle cell (SMC) specific markers both at the mRNA and protein levels. Gelatin coating could substantially enhance DPSC differentiation potential than Norm-c-induced cells. However, the absence of mature marker MHY-11 by immunostaining results from all treatment groups further indicated the development of immature and synthetic SMLCs. Finally, it was concluded that DPSC differentiation ability into SMLCs can be enhanced under cytokine treatment as well as by altering the cellular niche by precoating the culturing plates with suitable substrates. However, to get fully functional, contractile, and mature SMLCs, still many different cytokine cocktail combinations and more suitable coating substrates will be needed.

## 1. Introduction

Mesenchymal stem cells (MSCs) are the specialized cells which can be easily isolated from different tissues of the body and can self-replicate, propagated in vitro to desired cell numbers, having multidifferentiation potential and immunomodulatory features and showing better homing abilities when transplanted in vivo [1]. Due to such peculiar characteristics, MSCs have been successfully used to treat neurodegenerative, cardiac, pancreatic, and hepatic disorders [2–4]. However, the selection of a suitable MSC source is also highly important as different source-derived stem cells have been shown to display varied stemness and differentiation abilities [5–7]. A number of discarded tissues obtained as a result of routine surgical procedures have also been given importance as valuable MSC sources due to their easy availability, issueless isolation procedures, and high cell yield. One such valuable MSC source is the dental tissue which possesses different types of MSC sources, namely, pulp, follicle, and papilla which can transdifferentiate into desirable lineages with functional abilities when induced under appropriate culturing conditions [6]. Interestingly, continuous efforts are in progress to improve the efficacy of these cells both in vitro and in vivo. Previously, our group has already verified the importance of single donor-derived different dental MSC types and demonstrated dental pulp-derived MSCs (DPSCs) to be the best MSC source among others [6]. Focusing on cellular niche by using more appropriate extracellular matrix (ECM) components (mimicking in situ conditions) can also promote in vitro differentiation ability of the MSCs to greater extent. Therefore, in the present study, dental pulp-derived MSCs (DPSCs) were evaluated for their differentiation ability into smooth muscle-like cells (SMLCs) with special focus on the effect of suitable cytokines along with different coating substrates.

Isolated cells must possess characteristic features as per International Society for Cellular Therapy guidelines [8] to confirm their stemness. Moreover, isolated cells can also transdifferentiate into other lineages when induced under specific culturing conditions, undergo morphological alterations, and display positive expression for associated markers both at the mRNA and protein levels. However, it is not all the time that desired morphological changes have been shown by differentiated cells despite having adequate marker expression [9]. A lot more depends upon the selected MSC source as easy and issueless isolation, high cell yield, stemness features, and ability to respond appropriately under given inductions form the basis of selection and attainment of desirable results. Dental pulp-derived MSCs (DPSCs) were shown to have all such characteristics [6] and therefore targeted to get SMLCs. To get differentiated SMLCs, researchers have used MSCs from different sources such as Wharton's jelly, bone marrow, hair follicle, adipose, amniotic fluid, and dental pulp and induced them with suitable cytokines (mostly TGF-*β*) alone or in combination with other factors including PDGF-BB, BMP-4, and ascorbic acid [1, 10–14]. Moreover, different cytokine treatments have shown different extents of differentiation from different source-derived MSCs. Fewer studies have also demonstrated the use of conditioned media from cultured bladder-derived cells [14] or even used coculturing methods to get in vitro differentiated SMLCs [15]. But not many studies have evaluated the effect of coating substrates along with cytokine treatments to get differentiated SMLCs. Therefore, the present study is aimed at evaluating the differentiation potential of DPSCs towards SMLCs under the effect of coating substrate and suitable cytokines.

## 2. Materials and Methods

All chemicals were purchased from Sigma chemical company (Sigma, St. Louis, MO, USA) and media from Gibco (Invitrogen, Burlington, ON, Canada), unless otherwise specified.

### 2.1. Isolation and Culturing of DPSCs

Dental pulp tissue samples were obtained from the patients undergoing wisdom tooth extraction at Gyeongsang National University Changwon Hospital (GNUCH). After getting donor's consent and approval from the committee for clinical research at GNUCH (GNUCH-2018-11-002), samples were aseptically transported to the laboratory within 3-4 hours. Dental pulp-derived cells were isolated according to the previously published protocol [16]. Briefly, dental pulp tissues (*n* = 5) were carefully separated from the extracted wisdom tooth using a sterile scalpel, gently washed several times with 1% penicillin-streptomycin (10,000 IU and 10,000 *μ*g/ml, respectively; Pen-Step, Gibco Life Technologies) containing sterile Dulbecco's phosphate-buffered saline (DPBS), and then minced into small pieces using fine surgical blades. Furthermore, chopped tissue pieces were enzymatically digested with 0.1% collagenase type IV for up to 1 hour at 37°C while vortexing after every 15 minutes. Enzyme inactivation was stopped by adding 10% fetal bovine serum (FBS, Gibco Life Technologies) containing advanced Dulbecco's modified Eagle's medium (ADMEM, Gibco, Life Technologies), and the mixture was filtered through 100 *μ*m and 40 *μ*m cell strainers (BD Falcon, Franklin Lakes, NJ, USA) followed by centrifugation at 300 × g for 5 minutes. Finally, supernatant was discarded and pellet was substituted with the ADMEM and cultured onto cell culture plates (Nunc™, Roskilde, Denmark). Cell culture plates were kept inside a humidified 5% CO_2_ incubator at 37°C for promoting the cell growth in vitro. Cells started forming colonies within 2-3 days and attained confluency (~80%) within 7-8 days. At this stage, cell harvesting was done using 0.25% (*w*/*v*) trypsin-EDTA (Gibco), followed by centrifugation at 300 × g for 5 minutes and the cells were further subcultured up to the third passage. Morphological evaluations were performed using an inverted phase contrast microscope (Nikon DIAPHOT 300, Japan). Media was changed every alternative day.

### 2.2. Cell Surface Marker Expression

Using flow cytometry (BD FACSCalibur; Becton Dickinson and Company, Franklin Lakes, NJ, USA), third passage DPSCs were evaluated for the expression of cell surface marker expression according to the previously described protocol [7]. Briefly, cells (~80% confluence) were harvested and fixed with 3.7% formaldehyde solution for 30 minutes. After washing thrice with DPBS, cells were directly labelled with FITC-unconjugated anti-mouse HLA-DR (1 : 100; BD Pharmingen), anti-mouse CD14 (1 : 100; BD Pharmingen), and anti-mouse CD19 (1 : 100; BD Pharmingen) and FITC-conjugated mouse anti-human CD90 (1 : 100; BD Pharmingen, BD Bioscience, NJ, USA), anti-mouse CD44 (1 : 100; BD Pharmingen), mouse anti-human CD34 (1 : 100; BD Pharmingen), anti-mouse CD105 (1 : 100; BD Pharmingen), mouse anti-human CD45 (1 : 100; BD Pharmingen), and anti-mouse CD73 (1 : 100; BD Pharmingen) for 1 hour at 4°C. For vimentin expression, 3.4% formaldehyde fixed cells were first permeabilized with 0.1% Triton-X (Sigma-Aldrich) and then incubated with primary mouse anti-vimentin antibody (1 : 100; Sigma-Aldrich) and finally incubated with FITC-conjugated goat anti-mouse secondary antibody (1 : 100; Sigma-Aldrich). To remove the excess and unbounded antibodies and to avoid mixing, incubated cells were thoroughly washed with DPBS (4-5 times) at every sequential step. FITC-conjugated anti-mouse IgG was used as isotype control. All experiments were performed in triplicates.

### 2.3. In Vitro Differentiation Potential towards Mesenchymal Lineages

DPSCs at passage 3 were seeded onto 6-well culturing plates for evaluating their in vitro potential to differentiate into mesenchymal lineages. Cells at 70% confluence were induced to adipogenic and osteogenic lineages by culturing under lineage-specific conditions for 21 days following previously published protocol [7]. For adipogenic differentiation, DPSCs were induced with adipogenic specific media consisted of 10% ADMEM supplemented with 1 mM dexamethasone, 100 mM indomethacin, 10 mM insulin, and 500 mM isobutyl methyl xanthine. To confirm the successful differentiation, induced cells were stained with Oil red O solution to check the accumulation of lipid droplets (hallmark of differentiated adipocytes). Differentiated cells were also evaluated for the lineage-specific marker expression (*CEBPα*, *PPAR-γ*, and *FABP*) using real-time polymerase chain reaction (RT-PCR). For osteogenic differentiation, cells were induced with osteogenic lineage-specific media consisted of 10% ADMEM supplemented with 0.1 mM dexamethasone, 10 mM glycerol-2-phosphate, and 50 mM ascorbate-2-phosphate. Furthermore, differentiated cells were stained with Alizarin red and von Kossa for confirming the successful differentiation. The extent of differentiation was also evaluated by checking the expression of osteogenic specific markers (*ON*, *BMP2*, and *RUNX2*). Untreated cells were taken as control. Media was changed after every two days' interval.

### 2.4. Vascular Smooth Muscle-Like Cell Differentiation

To evaluate the effect of coating substrate and cytokine treatment regarding SMLC differentiation, both the commercially used normal (Nunc™, Roskilde, Denmark) (Norm-c) and 0.1% gelatin-precoated (Gel-c) 6-well plates were used to culture the cells and were further induced under widely used cytokines, namely, TGF-*β*1 and PDGF-BB. To differentiate DPSCs into SMLCs, previously used protocols with slight modifications were used [1]. Briefly, 5 ng/ml of TGF-*β*1 and 2 ng/ml of PDGF-BB were used to treat the cells at different time intervals, i.e., day 7, day 14, and day 21. Dulbecco's modified Eagle's medium (DMEM) supplemented with 10% fetal bovine serum (FBS) was used to culture both control and treated cells. Untreated DPSCs cultured onto normal culturing plates (without 0.1% gelatin precoating) were taken as control. Time-dependent morphological alterations were evaluated under an inverted phase contrast microscope (Nikon DIAPHOT 300, Japan), and images were taken accordingly. Harvested cells at different time intervals were evaluated for smooth muscle specific marker expression both at the mRNA and protein levels. Cells seeded onto 0.1% gelatin-coated plates were also used to evaluate the effect of gelatin coating alone (without using cytokines) regarding smooth muscle specific marker's mRNA expression. Media was changed twice in a week.

### 2.5. Collagen Gel Contraction Assay

The functional ability of the differentiated cells was checked by collagen gel contraction assay according to previously published protocol [17]. Briefly, a cell collagen suspension was created by mixing an aliquot of 1 × 10^5^ cells with soluble rat tail collagen type 1 (Gibco, Life Technologies) added with NaOH (1 M; Sigma), acetic acid, and DMEM 10x mixture. A total of 500 *μ*l of cell collagen suspension mixture was placed onto a 12-well tissue culture plate followed by polymerization for 20-30 minutes at room temperature. After that, dissociation of polymerized cells was done with the help of a 200 *μ*l pipette tip and 500 *μ*l DMEM was added in each well to promote the growth of the cells. Plates were kept inside a humidified 5% CO_2_ incubator at 37°C. To confirm the extent of gel contraction by the differentiated cells, gel lattice diameter was measured before and after mechanical release of the cell collagen lattice for contractile force measurement. Release of cell deposited lattices was performed by administering 60 mM KCl as agonist in serum-free media. Change in the diameter of collagen lattices from all the control and differentiated DPSCs (Norm-c and Gel-c) was evaluated at different time intervals, i.e., 4 hours and 8 hours. Finally, for the evaluation of the extent of contraction, values were calculated by using the formula (*D*_u_ − *D*_r_)/*D*_u_ × 100, where *D*_u_ and *D*_r_ represent the diameter unreleased and released lattices, respectively. The same method was repeated with undifferentiated DPSCs and taken as control. The experiment was performed in triplicates.

### 2.6. Immunocytochemistry

For immunocytochemical staining, both the control and differentiated cells were firstly fixed with 4% formaldehyde solution for 1 hour and permeabilized with 0.2% Triton X-100 for 15 minutes at room temperature. Both types of cells were further blocked for 1 hour with 1% bovine serum albumin (BSA) containing DPBS followed by overnight incubation at 4°C with primary antibodies such as rabbit monoclonal anti-alpha smooth muscle-22 (SM-22, 1 : 250, Abcam), mouse monoclonal anti-alpha smooth muscle actin (*α*-SMA, 1 : 250, Abcam), rabbit monoclonal anti-calponin (Calponin, 1 : 250, Abcam), rabbit-polyclonal anti-smoothelin, and rabbit polyclonal anti-myosin heavy chain 11 (MHY-11, 1 : 250, Abcam). To remove extra or unbounded primary antibody, incubated cells were washed with DPBS for at least 4-5 times following gentle agitation. Furthermore, cells were incubated with donkey anti-mouse IgG FITC (1 : 4000, Santa Cruz Biotechnology) and goat anti-rabbit IgG CFL 488 (1 : 4000, Santa Cruz Biotechnology) secondary antibodies for 1 hour at 37°C, respectively. Finally, after being washed with DPBS for 4-5 minutes, cell nuclei were counterstained with 1 *μ*g/ml of 4′,6-diamidino-2-phenylindole (DAPI) for 5-7 minutes at room temperature and images were taken using a fluorescent microscope (Leica, Wetzlar, Germany). To eliminate the background autofluorescence, control cells were directly stained with corresponding secondary antibodies. Immunostaining results from all the experimental groups in relation to SMC specific markers were quantified by using ImageJ v1.52a software.

### 2.7. Western Blotting

Dental pulp-derived MSCs were evaluated for pluripotency-associated marker's protein expression using western blotting. With the help of protease inhibitor containing RIPA buffer (Thermo Scientific, Rockford, IL, USA), protein lysate was prepared from treated and untreated control cells and protein concentration was estimated using a Microplate BCA Protein Assay Kit (Pierce Biotechnology, Rockford, IL, USA). After separating a total of 25 *μ*g of protein sample with the 12% sodium dodecyl sulfate-polyacrylamide gel electrophoresis (SDS-PAGE, Mini Protean, Bio-Rad, Hercules, CA, USA), protein-loaded gels were transferred onto polyvinylidene difluoride membranes (PVDF, Millipore, USA). Furthermore, membrane incubation was done with primary antibodies such as goat anti-Oct-3/4 (1 : 250, Santa Cruz Biotechnology), rabbit anti-Sox-2 (1 : 250, Santa Cruz Biotechnology), and goat anti-Nanog (1 : 250, Santa Cruz Biotechnology) for overnight at 4°C. Unbounded antibodies were removed by washing 4-5 times (5 minutes each wash) with TBST, and blots were further incubated with horseradish peroxidase- (HRP-) conjugated goat anti-rabbit IgG (1 : 10,000, Santa Cruz Biotechnology) and donkey anti-goat IgG (1 : 10,000, Santa Cruz Biotechnology) secondary antibodies at room temperature for 1 hour. Finally, enhanced chemiluminescence (ECL; SuperSignal, West Pico Chemiluminescent Substrate, Pierce, IL, USA) was used for the immunoreactivity detection and blots were exposed to X-ray films.

### 2.8. Real-Time Polymerase Chain Reaction (RT-PCR)

Both the control and treated cells harvested at different intervals of time (day 7, day 14, and day 21) were used for RNA isolation using the RNeasy mini kit (Qiagen, Valencia, CA, USA) following the manufacturer's guidelines with the additional elimination of genomic DNA. For evaluation of concentration and purity of the total RNA, optical density measurement was performed at 260 nm and 260 nm/280 nm ratio. Furthermore, using a total of 2 *μ*g RNA, complementary DNA (cDNA) was prepared using the Omniscript RT kit (Qiagen) with oligo-dT primer, and the reaction was carried out at 37°C for 60 minutes. To evaluate the expression of pluripotency (*Oct4*, *Sox2*, and *Nanog*) and smooth muscle specific markers (*SM22α*, *αSMA*, *calponin* (*CALP*), *smoothelin* (*SMTN*), and *myosin heavy chain II* (*MHY-II*)), real-time PCR (RT-PCR) was carried out on a Rotor Gene Q (Qiagen) using the Rotor Gene™ SYBR Green PCR kit (Qiagen). For data normalization, tyrosine 3-monooxygenase/tryptophan 5-monooxygenase activation protein zeta (YWHAZ) was used as a housekeeping gene. Sample (25 *μ*l reaction volume) included mixing of 50 ng cDNA with 10 *μ*l SYBR Green Mix, 2 *μ*l each of forward and reverse primers (400 nM concentration) along with 4 *μ*l of RNase-free water. Finally, RT-PCR assay was performed as per the manufacturer's instructions. RT-PCR settings included initial denaturation at 95°C for 2 minutes, followed by 40 PCR cycles of 95°C for 5 seconds, 60°C for 10 seconds followed by melting curve from 60°C to 95°C at 1°C/second and then cooling at 40°C for 30 seconds. To analyze the CT values and melting curve of each sample, Rotor-Gene Q software (Qiagen) was used. Relative level of mRNA expression was calculated by using 2^-*ΔΔ*Ct^, whereas 2% agarose gel electrophoresis was performed to evaluate the PCR products and furthermore zoom browser EX5.7 software (Canon) was used to analyze the gel images. The list of primers used is shown in Table 1. All experiments were performed in triplicates.

### 2.9. Statistical Analysis

All experiments were performed in triplicates and were analyzed for their statistical differences by Student's *t*-test and one-way ANOVA using SPSS 21.0. Data was reported as the mean ± standard error (SE). For multiple comparisons, Tukey's test was performed. Values were considered significant when *p* < 0.05.

## 3. Results

### 3.1. Morphological Evaluation and Pluripotency Marker Expression

**Figure 1:**
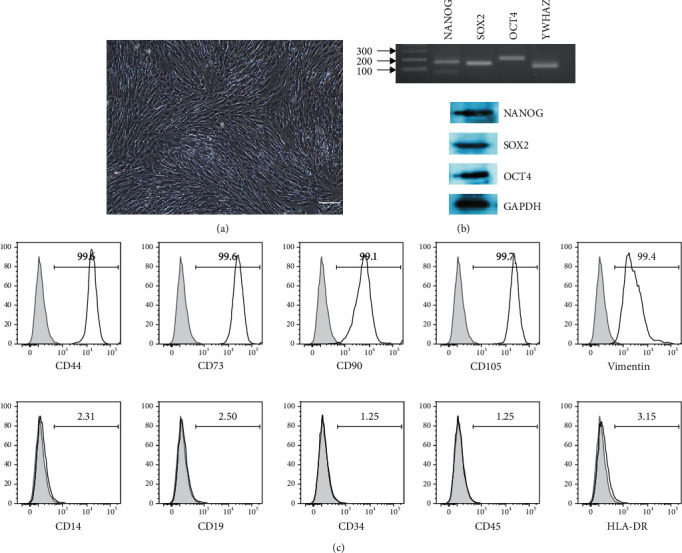
Morphological observations, pluripotency, and cell surface marker expression. DPSCs displayed (a) fibroblastoid morphology (scale bar = 100 *μ*m) and also showed (b) positive expression for pluripotency specific OCT4, SOX2, and NANOG markers. (c) Isolated cell's stemness was further confirmed by the presence of mesenchymal specific cell surface markers, i.e., CD44, CD73, CD90, CD105, and vimentin while lacking expression for CD14, CD19, CD34, CD45, and HLA-DR. Graph showing percentage of cells having positive and negative cell surface marker expression.

After being aseptically transported from the hospital, the extracted third molars were used to isolate dental pulp cells using enzymatic isolation method. Cells showed firm attachment with the plastic surface (culturing plate) and started showing colony formation within 5-6 days and displayed typical fibroblast morphology (Figure 1(a)). At 80% confluence, cells were further subcultured up to passage 3 to get homogenous population of the cells. Cells at passage 3 were used for the whole experimentation. Cells were further confirmed for their stemness characteristics by evaluating the expression of pluripotency specific markers, namely, OCT4, SOX2, and NANOG both at the mRNA and protein levels (Figure 1(b)).

### 3.2. Cellular Phenotyping

To confirm the expression of MSC specific cell surface markers, isolated dental tissue-derived cells were evaluated by flow cytometry. DPSCs displayed strong expression for the positive mesenchymal markers including CD44, CD73, CD90, CD105, and vimentin whereas they displayed negative expression for CD14, CD19, CD34, CD45, and HLA-DR (Figure 1(c)). Experiments were performed in triplicates, and no significant differences were seen in any cell line regarding the expression of MSC specific positive and negative CD markers.

### 3.3. Multilineage Differentiation Potential

**Figure 2:**
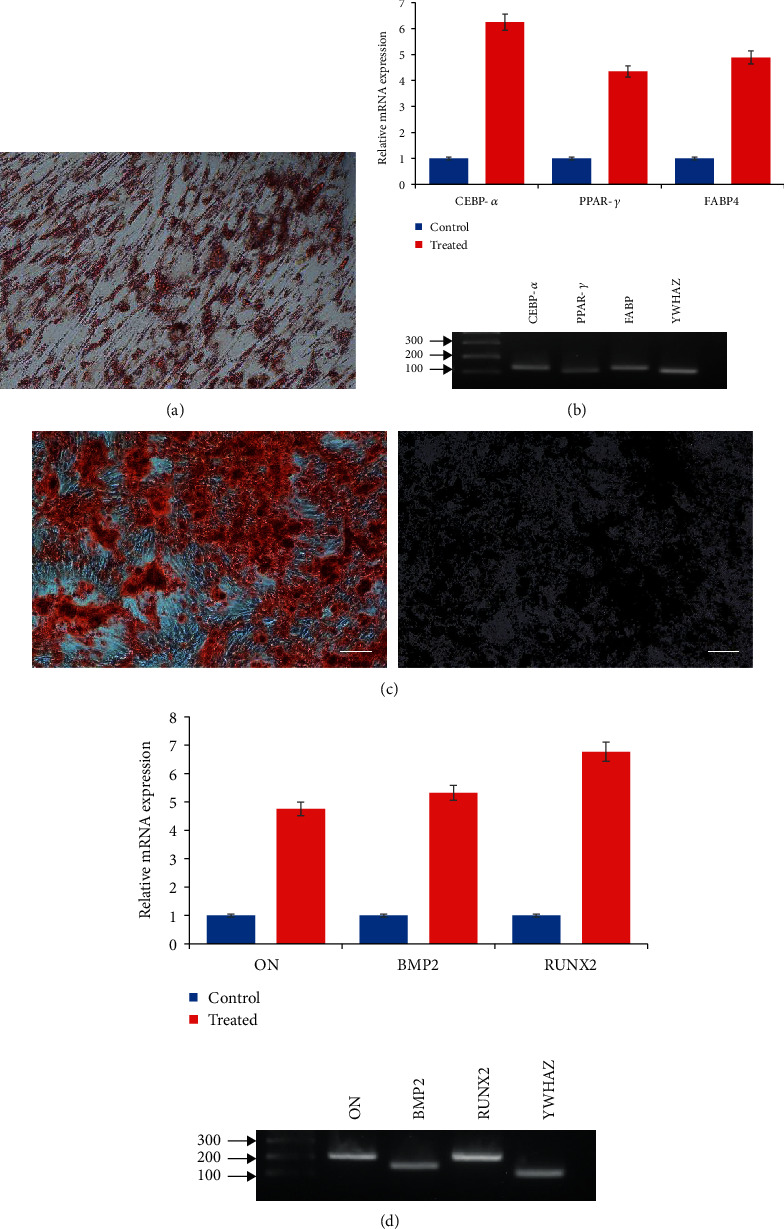
Adipogenic and osteogenic differentiation potential. DPSCs showed their differentiation ability to develop adipocytes and osteocytes when induced under specific culturing conditions. (a) Confirmation of successful adipocyte differentiation by positive Oil red O staining depicting development of intracellular lipid droplets. Differentiated adipocytes showed higher expression for *CEBP-α*, *PPAR-γ*, and *FABP4* markers. (b) Positive Alizarin red and von Kossa staining by differentiated osteocytes. Differentiated cells showed higher expression for osteocyte specific *ON*, *BMP2*, and *RUNX2* markers.

Multilineage differentiation potential of the isolated cells was confirmed by inducing the DPSCs into adipocyte- and osteocyte-specific culturing conditions for 21 days. Cells were successfully differentiated into adipocyte and osteocyte lineages and displayed positive expression of corresponding stains and marker expression. Adipocyte differentiation was confirmed by the successful Oil red O stain by the intracellular lipid droplets, which is the hallmark of differentiated adipocytes (Figure 2(a)). Differentiated cells also displayed positive expression of *CEBP-α*, *PPAR-γ*, and *FABP4* genes (Figure 2(b)). Osteocyte differentiation was evaluated by the positive staining of the differentiated cells by Alizarin red and von Kossa stains which confirmed the nodule formation (Figure 2(c)). Differentiated osteocytes were also shown to have positive expression of *ON*, *BMP2*, and *RUNX2* (Figure 2(d)). No such developmental changes were shown by undifferentiated control cells. No significant differences were observed by any of the isolated DPSC line when performed in triplicates.

### 3.4. Smooth Muscle-Like Cell Differentiation under Cytokine Induction and Different Culturing Plates

**Figure 3:**
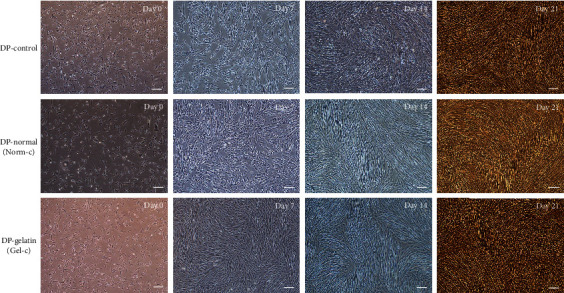
Smooth muscle-like cell differentiation potential: morphological alterations and smooth muscle specific marker expression. Phase contrast microscopic images from control and induced DPSCs at different time intervals; scale bar = 100 *μ*m.

**Figure 4:**
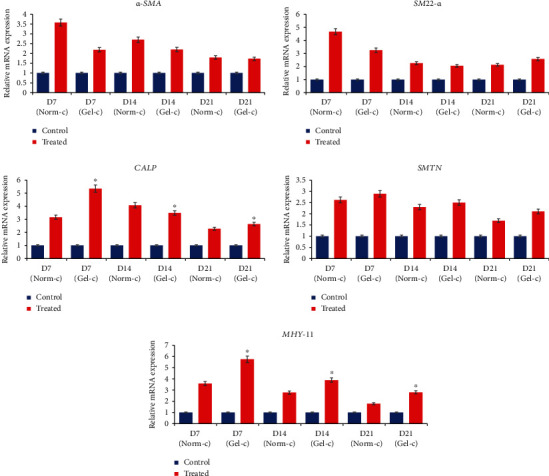
DPSCs induced under different treatments (Norm-c and Gel-c) were shown to have positive expression for SMC specific early (*α-SMA* and *SM22-α*), mid (*CALP*), and late (*SMTN* and *MHY-11*) markers. Comparatively higher expression was shown by differentiated cells in comparison to untreated control. The Norm-c and Gel-c treatment groups did not show any significant differences for *α-SM*, *SM22-α*, and *SMTN* whereas the Gel-c treatment group displayed significantly higher expression for *CALP* and *MHY-11*. Significant differences were considered when *p* < 0.5. ^∗^Significant difference (*p* < 0.05) between the samples.

**Figure 5:**
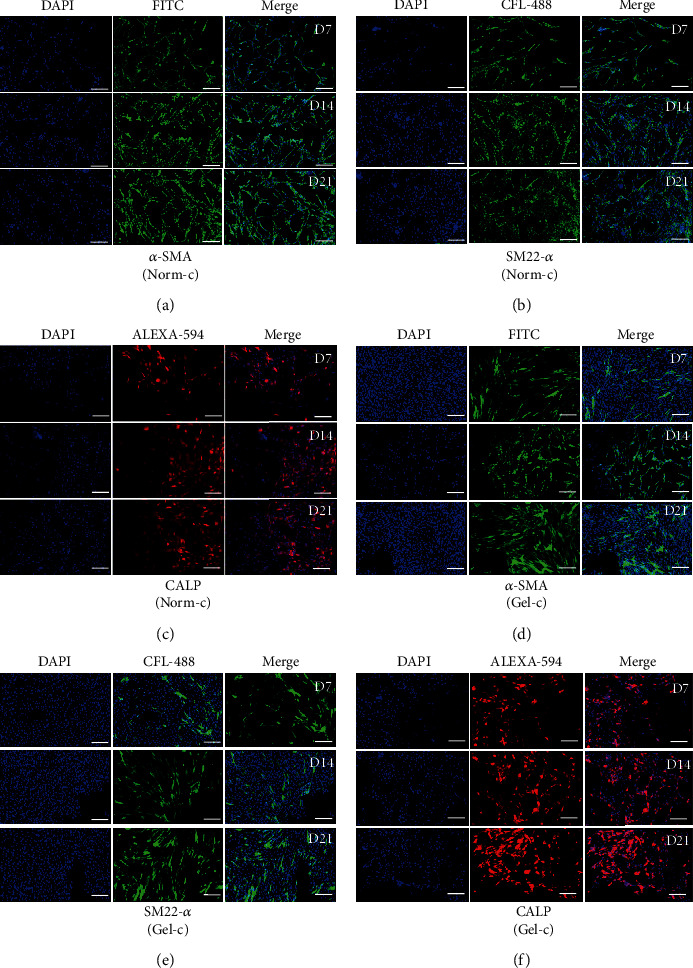
Immunocytochemical analysis of differentiated DPSCs induced under different treatments. (a–f) DPSCs induced under different treatments were analyzed for SMC specific markers. All treatment groups were shown to have positive expression for early (*α*-SMA and SM22-*α*) and mid (CALP) markers without any significant differences; scale bar = 100 *μ*m.

**Figure 6:**
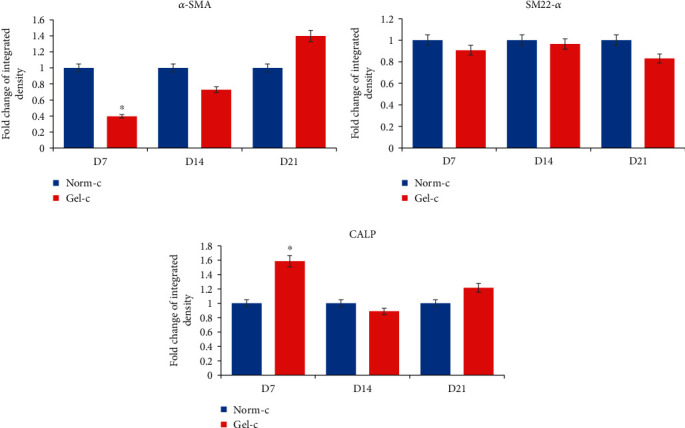
Quantification of positively immunostained differentiated MSCs. All the differentiated MSCs under both the treatment groups (Norm-c and Gel-c) showing positive immunostaining results were quantified using ImageJ v1.52a software. A significant difference was observed in fold change of integrated density by day 7 Gel-c and Norm-c treated MSCs in relation to *α*-SMA and CALP markers. However, no significant difference was observed among any other group for any marker in any time duration. Significant differences were considered when *p* < 0.5. ^∗^Significant difference (*p* < 0.05) between the samples.

Both of the treatment groups (i.e., Norm-c and Gel-c) showed elongated fibroblastoid morphology with slight alterations, but typical smooth muscle specific fusiform shapes were absent (Figure 3). However, MSCs (without any cytokine induction) cultured on normal culturing plates and 0.1% gelatin-precoated plates did not show any morphological differences. Differentiated cells from both the treatment groups displayed positive expression for smooth muscle specific early, mid, and late markers when evaluated by RT-PCR (Figure 4). For the expression of early markers, i.e., *α-SMA* and *SM22-α*, as well as mature marker *SMTN*, no significant differences were observed among both the Norm-c and Gel-c treatment groups whereas DPSCs induced with cytokines using 0.1% gelatin-precoated plates showed significantly higher expression for mid (*CALP*) and mature SMC marker (*MHY-11*) when compared to Norm-c differentiated cells. However, a time-dependent gradual decrease in the expression of all markers was seen in all the treatment groups. The effect of 0.1% gelatin precoating (Gel-c UT, i.e., without cytokines) onto SMLC differentiation potential in comparison to the untreated control group (non-precoated and without any cytokine induction) and 0.1% gelatin precoating followed by cytokine induction group (Gel-c TRT, i.e., with cytokines) was also evaluated. A marginal increase (no significant difference) in the marker expression under Gel-c UT condition was observed than the untreated control group (Supplementary Figure 1). Therefore, untreated MSCs propagated onto 0.1% gelatin-precoated plates were neglected for further experimentation. Interestingly, the expression level was elevated when 0.1% gelatin-precoated plates were induced with cytokines. Induced cells were also evaluated for their time-dependent protein expression using immunocytochemistry (Figures 5(a)–5(f)). Both the differentiated groups showed positive expression for SMC specific early (*α*-SMA and SM22-*α*) and mid (CALP) markers. However, a marginal SMTN expression was occasionally shown (when evaluated from experiments performed in triplicates) by both the treatment groups (data not shown). Additionally, none of the treatment groups were shown to have positive expression for mature marker MHY-11. Immunocytochemical staining results from all the experimental groups targeting SMC specific markers were quantified as fold change of integrated density using ImageJ v1.52a software, and data was graphically presented in a time-dependent manner (Figure 6). Except *α*-SMA and CALP markers at day 7 from Norm-c and Gel-c treated MSCs, no statistical differences were observed among other markers in any time duration. Control untreated cells did not display positive expression for any marker.

### 3.5. Collagen Gel Lattice Assay

**Figure 7:**
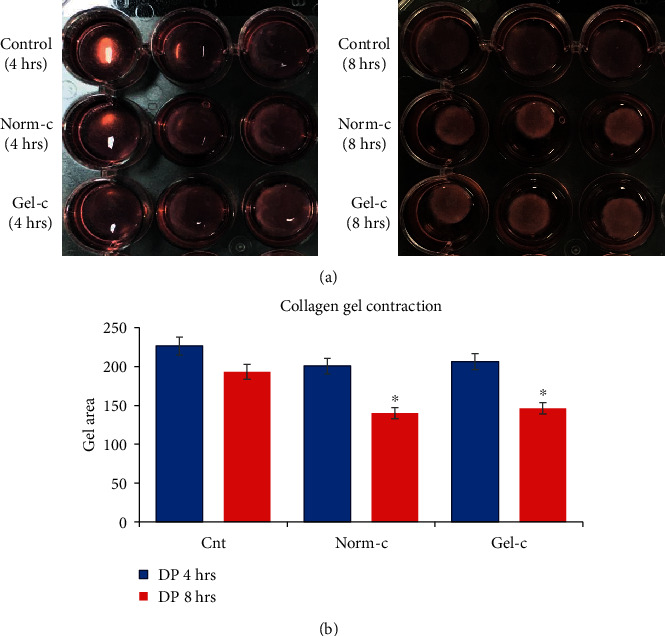
Collagen gel contraction ability by differentiated DPSCs. (a, b) Both the untreated and treated DPSCs were evaluated for their ability to contract the collagen gel lattices under KCl (60 mM) stimulation. In a time-dependent manner, both the treatment groups (Norm-c and Gel-c) showed higher gel contraction ability than their untreated counterparts. There were no significant differences among both the treatment groups. ^∗^Significant difference (*p* < 0.05) between the samples.

Differentiated cells from both of the experimental groups showed time-dependent enhanced contraction ability upon KCl stimulation in comparison to their control uninduced (no KCl induction) counterparts. Interestingly, Norm-c and Gel-c treatment groups did not show any significant differences among their ability to contract collagen gels. However, the control groups corresponding to undifferentiated cells also showed noticeable marginal gel contraction ability but at much lower levels than differentiated cells (Figures 7(a) and 7(b)).

## 4. Discussion

Smooth muscles are contractile cells which play an important role in the proper functioning of many vital organ/organ systems such as respiratory, vascular, gastrointestinal, genitourinary, and stomach [18]. SMCs also help in vasoconstrictions and vasodilation and thereby regulate blood vessel diameter [19, 20]. Any abnormalities in these smooth muscle cells can ultimately lead to impaired functioning of the associated organs. Under such conditions, contractile SMCs get transformed to synthetic phenotypes while undergoing high proliferation, reduced expression of contractile proteins, and extensive extracellular matrix synthesis [19–21]. Such abnormalities if not treated at proper time may result in more severe complications such as hypertension, arteriosclerosis, aneurysm, and restenosis. Stem cell-based cell therapy can be a useful, highly efficient, and curative measure to treat many of such disorders. Since many years, researchers have efficiently succeeded in isolating MSCs from different sources and characterized them as per ISCT guidelines [8]. The presence of pluripotency specific markers (OCT4, SOX2, and NANOG), cell surface marker profiling, and multilineage differentiation potential (adipogenesis, osteogenesis, and chondrogenesis) have been considered as gold standards to evaluate the extent of stemness features present in the isolated cells. Evaluation of these minimal features has been the basis for selecting cells for in vitro and in vivo experimental purposes. DPSCs displayed high stemness features in accordance with the previously published reports [6, 16].

Along with the ability to get differentiated into mesenchymal cell lineages (adipocytes and osteocytes), DPSCs were evaluated for the expression of widely reported positive cell surface markers (CD44, CD73, CD90, and CD105) as well as negative markers (CD34 and CD45). DPSCs also showed higher positivity for vimentin. Moreover, isolated cells were also evaluated for other valuable hematopoietic markers including CD14, CD19, and HLA-DR whose negative expression is a prerequisite for avoiding immunity-associated rejections during allogenic transplantation studies [22]. After confirming stemness, researchers have focused on assessing the transdifferentiation ability of isolated MSCs under specific cytokine treatments. As far as SMLC differentiation is concerned, our group and researchers from different laboratories have successfully developed many efficient protocols using various cytokines to get in vitro differentiated SMLCs [1, 10–14]. However, not much focus has been given to the extracellular matrix or “cellular niche” which is equally important to provide conducive environment. The same phenomenon has been used to culture highly specialized cells such as embryonic stem cells and induced pluripotent cells which have been shown to display desired growth characteristics when cultured on specific coating substrates including 0.1% gelatin [23]. Therefore, the main focus of this study was to assess the effect of widely used cytokines along with different culturing environments on SMLC differentiation using DPSCs. To derive in vitro differentiated SMLCs, a number of studies have reported the use of TGF-*β*1 as a main differentiation inducer to get SMLCs in vitro, as it can alone direct MSCs to undergo SMC differentiation while attaining desired morphological alterations along with positive expression of relevant markers (both at the mRNA and protein levels) and functional competence [14, 24]. TGF-*β*1 in combination with other cytokines has also shown enhanced in vitro SMLC differentiation potential [11–13]. Moreover, our previous study has also demonstrated that TGF-*β*1 alone can induce Wharton's jelly MSCs to become SMLCs and the extent of differentiation can be further enhanced when induction media is supplemented with a cocktail combination of both TGF-*β*1 and PDGF-BB [1]. Therefore, we utilized this type of cytokine combination targeting another MSC source, i.e., DPSCs.

It is worth noticing that, despite having muscle cell specific differentiation promoting effects of PDGF-BB, it has also been shown to limit myogenic differentiation by acting as a negative regulator of SMC differentiation [10, 25]. Similar findings were shown by Wanjare and colleagues [26] who observed the development of synthetic vascular smooth muscle-like cells upon treating human pluripotent stem cell lines with high serum with PDGF-BB and TGF-*β*1. Interestingly, a matured contractile vascular smooth muscle cell phenotype was observed in the induced cells when induction conductions were changed with serum starvation and PDGF-BB deprivation. Keeping these research outputs in mind, it was assumed that lower concentration of PDGF-BB in combination with main myogenic inducer “TGF-*β*1” can promote SMC differentiation more efficiently without pausing any adverse effects. Therefore, DPSCs were induced with higher TGF-*β*1 concentration (5 ng/ml) and lower PDGF-BB (2 ng/ml) using different culturing plates (Norm-c and Gel-c). In accordance with previously published reports, induced cells showed positive expression for SMC specific early, mid, and late markers [1, 13]. DPSCs induced under different culturing environments were shown to have similar expression levels for markers including *α-SMA*, *SM22-α*, and *SMTN*. However, Gel-c differentiated cells displayed significantly higher mRNA expression levels for *CALP* and *MHY-11* than Norm-c-induced cells. Altering the culturing conditions by providing conducible cellular niche by means of gelatin coating may have resulted in higher expression level. However, to elaborate the actual mechanism behind promoting effects by gelatin coating will need advanced experimental procedures in the future. On the other hand, overall fold change expression from both the treatment groups was low than expected levels and they showed gradually declined expression patterns when evaluated in a time-dependent manner. The main reason behind such expression pattern is not known and will need further elucidation in the future. Like RT-PCR results, induced DPSCs (under both culturing conditions) displayed positive immunostaining results for *α*-SMA, SM22-*α*, and CALP (Figure 5), inconsistently expressed SMTN, but could not show positive staining results for main matured marker MHY-11 (data not shown). These results indicate that differentiated SMLCs were not properly matured and were having synthetic phenotype as shown by previously published reports [27]. Furthermore, differentiated cells from both the treatment groups were assessed for their ability to contract collagen gel lattices in a time-dependent manner. Consistent with other reports, differentiated DPSCs could successfully contract collagen gel lattices when stimulated with muscarinic receptors [10, 13]. Cells induced under both conditions (Norm-c and Gel-c) showed significantly higher gel contraction ability than untreated control cells. These results demonstrate the ability of the DPSCs to attain smooth muscle specific phenotype when induced under appropriate cytokines while culturing on suitable coating materials.

## 5. Conclusion

From the present study, it has been concluded that DPSCs have the ability to differentiate into SMLCs when induced under suitable culturing conditions. Although treatment with 5 ng/ml TGF-*β*1 and 2 ng/ml PDGF-BB could result into increased SMC specific marker expression and also assist in attaining collagen gel contraction ability, precoating with 0.1% gelatin could further enhance DPSCs to transdifferentiate into SMLCs but to a lesser extent than expected. However, to get fully functional matured and contractile SMLCs, still there is an immense need of elaborative research work focusing on using several cytokine cocktail concentrations with special emphasis on more suitable coating substrates to provide the most conducive cellular niche.

## Figures and Tables

**Table 1:** List of primers used to evaluate the marker (genes) expression profiling of DPSCs using RT-PCR.

Gene	Primer sequence	Product size (bp)	Accession no.
*OCT4*	F: AAGCAGCGACTATGCACAAC R: AGTACAGTGCAGTGAAGTGAGG	140	NM_002701.5
*SOX2*	F: CACCCACAGCAAATGACAGC R: AGTCCCCCAAAAAGAAGTCCAG	120	NM_003106.3
*NANOG*	F: GCAGATGCAAGAACTCTCCAAC R: CTGCGTCACACCATTGCTATTC	133	AB093576.1
*CEBP-α*	F: GAAGTCGGTGGACAAGAAC R: CATTGTCACTGGTCAGCTC	140	NM_004364.4
*PPAR-γ*	F: TTGCTGTCATTATTCTCAGT R: GAGGACTCAGGGTGGTTCAG	124	AB565476.1
*FABP4*	F: TGAGATTTCCTTCATACTGG R: TGGTTGATTTTCCATCCCAT	128	NM_001442.2
*ON*	F: GTGCAGAGGAAACCGAAGAG R: AAGTGGCAGGAAGAGTCGAA	202	J03040.1
*BMP2*	F: TAGACCTGTATCGCAGGCAC R: GGTTGTTTTCCCACTCGTTT	149	NM_001200.2
*RUNX-2*	F: ATGTGTGTTTGTTTCAGCAG R: TCCCTAAAGTCACTCGGTAT	199	NM_001024630.3
*α-SMA*	F: ACTGGGACGACATGGAAAAG R: CATACATGGCTGGGACATTG	168	BC093052.1
*SM22-α*	F: AGCCTTCTTTCCCCAGACAT R: CACCAGCTTGCTCAGAATCA	216	D17409.1
*CALP*	F: TGACCCCAAGAACCATATCC R: CAGGGACATGGAGGAGTTGT	169	BC141833.1
*SMTN*	F: CTGGGCAGTGTCACTCATGT R: CTGATCCAGCATCTTGTCCA	234	NM_001207018.2
*MHY-11*	F: CAGCCAGCATTAAGGAGGAG R: AAGTACCGCTCCCTCAGGTT	190	BC031040.1
*YWHAZ*	F: ACGAAGCTGAAGCAGGAGAAG R: TTTGTGGGACAGCATGGATG	111	BC108281.1

## Data Availability

Data access can be requested on demand with the corresponding author.

## Supplementary Materials

Supplementary Materials Supplementary Figure 1: comparative evaluation of the effect of different coating and noncoating culturing plates in association with or without cytokine induction regarding SMLC differentiation potential. In comparison to the control group, gelatin precoating (Gel-c UT; without cytokine induction) could marginally enhance (no significant difference) SMC specific marker expression. However, comparatively higher expression was seen when MSCs are cultured onto gelatin-precoated plates and induced with cytokines (Gel-c TRT; with cytokines). Significant differences were considered when *p* < 0.5. ^∗^Significant difference (*p* < 0.05) between the samples.
