# Supplementary material for: Human Dental Pulp-Derived Mesenchymal Stem Cell Potential to Differentiate into Smooth Muscle-Like Cells In Vitro
Source: Biomed Res Int. 2021 Jan 22;2021:8858412. doi: 10.1155/2021/8858412 (PMC7846403; doi:10.1155/2021/8858412)
Supplement: Supplementary Materials — Supplementary Figure 1: comparative evaluation of the effect of different coating and noncoating culturing plates in association with or without cytokine induction regarding SMLC differentiation potential. In comparison to the control group, gelatin precoating (Gel-c UT; without cytokine induction) could marginally enhance (no significant difference) SMC specific marker expression. However, comparatively higher expression was seen when MSCs are cultured onto gelatin-precoated plates and induced with cytokines (Gel-c TRT; with cytokines). Significant differences were considered when p < 0.5. ∗Significant difference (p < 0.05) between the samples. [file 8858412.f1.pptx]

## Slide 1
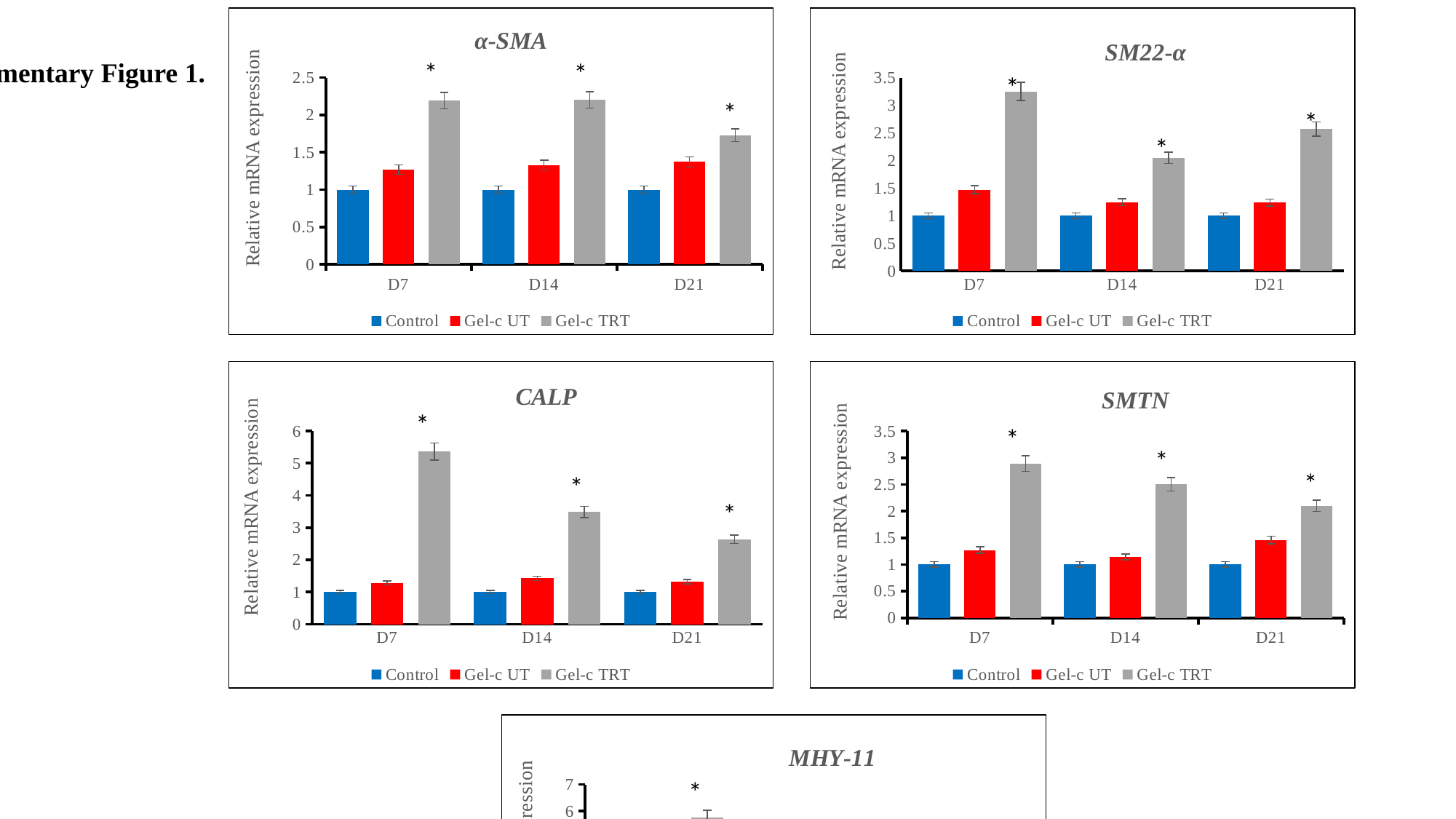

### Chart: α-SMA
| Category | Control | Gel-c UT | Gel-c TRT |
|---|---|---|---|
| D7 | 1.0 | 1.2677778621561526 | 2.19 |
| D14 | 1.0 | 1.3294625030829463 | 2.2 |
| D21 | 1.0 | 1.3723557179104633 | 1.73 |*
*
*
### Chart: SM22-α
| Category | Control | Gel-c UT | Gel-c TRT |
|---|---|---|---|
| D7 | 1.0 | 1.4694319462855805 | 3.25 |
| D14 | 1.0 | 1.2419377915072767 | 2.05 |
| D21 | 1.0 | 1.2380692333129961 | 2.57 |*
*
*
### Chart: CALP
| Category | Control | Gel-c UT | Gel-c TRT |
|---|---|---|---|
| D7 | 1.0 | 1.2862949924179352 | 5.36 |
| D14 | 1.0 | 1.4245748208719113 | 3.49 |
| D21 | 1.0 | 1.3196606781783828 | 2.64 |*
*
*
### Chart: SMTN
| Category | Control | Gel-c UT | Gel-c TRT |
|---|---|---|---|
| D7 | 1.0 | 1.27005174933861 | 2.89 |
| D14 | 1.0 | 1.1407921979900932 | 2.5 |
| D21 | 1.0 | 1.4580655909044336 | 2.1 |*
*
*
### Chart: MHY-11
| Category | Control | Gel-c UT | Gel-c TRT |
|---|---|---|---|
| D7 | 1.0 | 1.1863314634857938 | 5.75 |
| D14 | 1.0 | 1.32727785895523 | 3.89 |
| D21 | 1.0 | 1.3413149585793331 | 2.8 |*
*
*
Supplementary Figure 1.
